# Structural insights into Charcot–Marie–Tooth disease‐linked mutations in human GDAP1

**DOI:** 10.1002/2211-5463.13422

**Published:** 2022-05-20

**Authors:** Aleksi Sutinen, Giang Thi Tuyet Nguyen, Arne Raasakka, Gopinath Muruganandam, Remy Loris, Emil Ylikallio, Henna Tyynismaa, Luca Bartesaghi, Salla Ruskamo, Petri Kursula

**Affiliations:** ^1^ 6370 Faculty of Biochemistry and Molecular Medicine & Biocenter Oulu University of Oulu Finland; ^2^ Department of Biomedicine University of Bergen Norway; ^3^ 82219 VIB‐VUB Center for Structural Biology Vlaams Instituut voor Biotechnologie Brussels Belgium; ^4^ 70493 Structural Biology Brussels Department of Bioengineering Sciences Vrije Universiteit Brussel Belgium; ^5^ Stem Cells and Metabolism Research Program Faculty of Medicine University of Helsinki Finland; ^6^ Clinical Neurosciences, Neurology Helsinki University Hospital Finland; ^7^ Department of Neuroscience Karolinska Institutet Sweden

**Keywords:** Charcot–Marie–Tooth disease, GDAP1, GST superfamily, protein structure, neuropathy, stability

## Abstract

Charcot–Marie–Tooth disease (CMT) is the most common inherited peripheral polyneuropathy in humans, and its different subtypes are linked to mutations in dozens of different genes. Mutations in ganglioside‐induced differentiation‐associated protein 1 (GDAP1) cause two types of CMT, demyelinating CMT4A and axonal CMT2K. The GDAP1‐linked CMT genotypes are mainly missense point mutations. Despite clinical profiling and *in vivo* studies on the mutations, the etiology of GDAP1‐linked CMT is poorly understood. Here, we describe the biochemical and structural properties of the Finnish founding CMT2K mutation H123R and CMT2K‐linked R120W, both of which are autosomal dominant mutations. The disease variant proteins retain close to normal structure and solution behavior, but both present a significant decrease in thermal stability. Using GDAP1 variant crystal structures, we identify a side‐chain interaction network between helices ⍺3, ⍺6, and ⍺7, which is affected by CMT mutations, as well as a hinge in the long helix ⍺6, which is linked to structural flexibility. Structural analysis of GDAP1 indicates that CMT may arise from disruption of specific intra‐ and intermolecular interaction networks, leading to alterations in GDAP1 structure and stability, and, eventually, insufficient motor and sensory neuron function.

## Graphical abstract

Charcot–Marie–Tooth disease (CMT) is a hereditary neuropathy in humans, and GDAP1 is a common target for CMT‐linked mutations. Many of the mutation sites in the 3D structure of GDAP1 cluster into a region of intramolecular interactions. We studied in detail two of these mutations: H123R and R120W. We identify changes in mutant GDAP1 structure and stability.

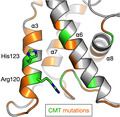

**Abbreviations**
CMT: Charcot–Marie–Tooth disease
GDAP1: ganglioside‐induced differentiation‐associated protein 1
GST: glutathione S‐transferase
MD: molecular dynamics
MOM: mitochondrial outer membrane
PNS: peripheral nervous system
rDRG: rat dorsal root ganglion
SAXS: small‐angle X‐ray scattering
SEC: size‐exclusion chromatography
SRCD: synchrotron radiation circular dichroism
TEV: tobacco etch virus

Inherited polyneuropathies are a genetically and clinically diverse group of neurodegenerative diseases affecting motor and sensory neurons in the peripheral nervous system (PNS) [1, 2]. Mutations in dozens of genes expressed in the PNS cause Charcot–Marie–Tooth disease (CMT). Based on clinical findings, CMT can be classified into three forms: demyelinating, axonal, and intermediate [3, 4]. The progress of CMT is linked to the hereditary pattern, whereby the autosomal recessive form has an earlier onset and more severe symptoms than the autosomal dominant form [5, 6, 7]. Understanding the molecular function of the proteins involved in the etiology of neuropathies is vital in efforts toward treatment and diagnosis.

Ganglioside‐induced differentiation‐associated protein 1 (GDAP1) is an integral mitochondrial outer membrane (MOM) protein, and the *GDAP1* gene is one of the most abundant targets for missense mutations linked to CMT [8, 9, 10]. Both autosomal dominant and recessive modes of inheritance are found, resulting in either autosomal recessive or dominant demyelinating CMT4, autosomal dominant axonal CMT2, or intermediate CMTRIA types of CMT, with varying phenotype severity [11]. The mutations R120W and H123R, which we focus on in this study, are both autosomal dominant mutations causing the CMT2K subtype. Both phenotypes show typical slow development after onset, and main symptoms include loss of sensation in limb extremities and muscle weakness. The clinical profiling of the phenotypes has been described earlier in Spain and Finland [12, 13, 14]. GDAP1 is ubiquitously expressed in tissues, but most of the expression confines to neuronal tissues [9, 15]. In the cell, GDAP1 localizes as a tail‐anchored MOM protein [16]. Structurally, GDAP1 resembles glutathione *S*‐transferases (GST), and it contains unique flexible loops [17, 18]. The most accurate structural data thus far cover the dimeric core GST‐like domain of human GDAP1, including the GDAP1‐specific insertion [18]. The transmembrane helix and the GST‐like domain are linked by a hydrophobic domain and possibly a flexible linker loop.

Glutathione *S*‐transferase superfamily members function in prokaryotic and eukaryotic metabolism through the utilization of reduced glutathione to catalyze a range of chemically diverse reactions. GSTs often contribute to mechanisms of neurodegenerative diseases [19, 20]. In comparison with many other enzyme superfamilies, GSTs are unique in that sequence conservation appears to be driven by fold stability instead of catalytic features, as reflected in the broad spectrum of GST substrates [21, 22]. While the function of GDAP1 is not fully understood at the molecular level, it has been linked to multiple mitochondrial events in neurons [23, 24], redox regulation, and signal transduction [25, 26].

In the Finnish population, the autosomal dominant founder mutation H123R accounts for as much as 20–30% of the local CMT cases [12, 27]. We carried out structural analysis of two selected autosomal dominant GDAP1 mutants, H123R and R120W, using X‐ray crystallography and complementary biophysical and computational techniques. In addition, we used three cell culture models, rat dorsal root ganglion (rDRG) neurons, human embryonic kidney 293 (HEK293T) cells, and human skin fibroblasts, to observe the oligomeric state of GDAP1 and the effects of the disease mutations therein.

## Methods

### Cloning

The GDAP1Δ295‐358 and GDAP1Δ303‐358 constructs used to produce soluble recombinant human GDAP1 in *E. coli* have been described [18]. Point mutations were generated in GDAP1Δ303‐358 by a site‐directed mutagenesis protocol with *Pfu* polymerase [28]. An N‐terminal His_6_‐affinity tag and a tobacco etch virus (TEV) protease digestion site were included in each construct. The full‐length GDAP1 coding sequence was subcloned into Gateway^®^ (Invitrogen) vectors pEN‐TTmcs and pSLIK‐HYGRO [29]. Point mutations were introduced as above. In addition to the *GDAP1* gene, the tetracycline‐responsive promoter element (tight‐TRE) was added within the cloning site [30], and a single N‐terminal FLAG‐tag was introduced into each construct [31]. All constructs were verified by DNA sequencing.

### Recombinant protein production

Soluble recombinant GDAPΔ295‐358 and GDAP1Δ303‐358 were expressed in *E*. *coli* BL21(DE3) strain using ZYM‐5052 autoinduction medium (24 h, 220 rpm, +37 °C) [32]. The cells were resuspended in binding buffer [40 mm HEPES, 400 mm NaCl, 2% glycerol, and 25 mm imidazole (pH 7.5)], containing an EDTA‐free protease inhibitor tablet (Sigma, Darmstadt, Germany), snap‐frozen in liquid nitrogen, and stored at −70 °C. Lysis of the cells was done by sonication, and the lysate was clarified by centrifugation (40 min, 30 600 *g*, +4 °C). The recombinant protein was captured on a Ni^2+^‐NTA HisPur^®^ affinity resin by gravity flow (Thermo Fisher Scientific, Waltham, MA, USA). Unbound proteins were washed with binding buffer. The matrix was eluted with the same buffer containing imidazole at 250 mm. The affinity tag was cleaved with TEV protease treatment in 25 mm HEPES, 300 mm NaCl, 2% (v/v) glycerol, and 1 mm TCEP in a dialysis tube (16 h, +4 °C). The His_6_‐tag and TEV protease were then removed by another Ni^2+^‐NTA affinity step. Size‐exclusion chromatography (SEC) was performed on a Superdex 75 10/300 GL increase column (Cytiva, Marlborough, MA, USA) using 25 mm HEPES (pH 7.5) and 300 mm NaCl (SEC buffer) as mobile phase.

For GDAPΔ295‐358, the Ni^2+^‐NTA purification protocol was identical, but 40 mm HEPES, 400 mm NaCl, 20 mm imidazole, and pH 7.5 were used as lysis and Ni‐NTA as washing buffer, and 32 mm HEPES, 320 mm NaCl, 500 mm imidazole, and pH 7.5 were used to elute bound proteins. EDTA‐free protease inhibitor cocktail (Roche) was included during cell freezing and lysis. TEV protease treatment was performed in dialysis against 40 mm HEPES, 400 mm NaCl, and pH 7.5 at +4 °C overnight, followed by a second Ni^2+^‐NTA affinity step. SEC was performed using a Superdex 200 16/60 HiLoad column (Cytiva) with 20 mm HEPES, 300 mm NaCl, 1% (v/v) glycerol, 0.5 mm TCEP, and pH 7.5 as mobile phase.

SEC peak fractions were analyzed with SDS‐PAGE, and Coomassie‐stained bands were used for protein identification using a Bruker UltrafleXtreme matrix‐assisted laser desorption/time‐of‐flight mass spectrometer (MALDI‐TOF‐MS). Tryptic peptides extracted from the gel were identified by a search in NBCI and Swiss‐Prot databases using biotools2.2 (Bruker, Billerica, MA, USA).

### Crystallization, data collection, and structure determination

Mutant GDAP1Δ303‐358 crystals were obtained using the sitting‐drop vapor‐diffusion method at +4 °C. Proteins were mixed with mother liquor on crystallization plates using a Mosquito LCP nanodispenser (SPT Labtech, Melbourn, UK). The protein concentration was 10–30 mg·mL^−1^ in 75 nL, and 150 nL of reservoir solution was added. H123R crystals were obtained in 0.15 m *DL*‐malic acid and 20% (w/v) PEG3350. R120W crystals were obtained in 0.1 m HEPES (pH 7.3) and 10% (w/v) PEG6000. Crystals were briefly soaked in a mixture containing 10% PEG200, 10% PEG400, and 30% glycerol for cryoprotection, before flash freezing in liquid N_2_.

A novel crystal form of wild‐type GDAP1Δ295‐358 was obtained at +8 °C in 200 mm NH_4_ formate, 25% (w/v) PEG3350 in a drop containing 150 nL of 8.64 mg·mL^−1^ protein, and 150 nL of reservoir solution. Cryoprotection was performed by adding 3 µL of cryoprotectant solution [75% (v/v) reservoir solution mixed with 25% (v/v) PEG200] directly into the crystallization drop, followed by crystal mounting and flash freezing with liquid N_2_.

Diffraction data collection at 100 K was conducted at the PETRA III synchrotron source (DESY, Hamburg, Germany) on the P11 beamline [33, 34] and the EMBL/DESY P13 beamline. Diffraction data were processed and scaled using XDS [35]. Crystal structures of wild‐type GDAP1Δ303‐358 [18] were used as search models in molecular replacement. Molecular replacement, model refinement, and structure validation were done using Phenix [36, 37] and CCP4 [38]. The models were refined using phenix.refine [39] and rebuilt using coot [40]. The structures were validated using molprobity [41]. The data processing and structure refinement statistics are shown in Table 1.

**Table 1:** Crystallographic data processing and structure refinement. Data for the highest‐resolution shell are shown in parentheses.

Protein	R120W GDAP1	H123R GDAP1	wtGDAP1
Data collection			
Beamline	P11/PETRA III	P11/PETRA III	P13/EMBL‐PETRA III
X‐ray wavelength (Å)	1.0332	1.0332	1.0332
Space group	P2_1_2_1_2_1_	P6_3_22	P3_1_21
Unit cell dimensions a, b, c (Å)	72.71, 115.88, 116.18	147.27, 147.27, 114.56	126.8, 126.8, 177.1
Resolution range (Å)	50–2.2 (2.3–2.2)	50–3.4 (3.5–3.4)	100–3.2 (3.39–3.20)
Completeness (%)	99.7 (98.8)	99.5 (98.6)	99.9 (99.9)
Redundancy	6.5 (6.7)	12.8 (12.6)	9.8 (9.9)
*R*_meas_ (%)	9.0 (191.3)	41.1 (339.8)	10.7 (387.4)
<*I*/σ*I*>	12.8 (1.0)	6.7 (0.9)	16.0 (0.6)
CC_1/2_ (%)	99.9 (69.6)	99.6 (60.5)	100.0 (17.7)
Wilson *B* (Å^2^)	49.4	104.5	129.4
Structure refinement			
*R*_cryst_/*R*_free_ (%)	21.1/23.3	25.0/29.1	25.1/27.6
RMSD bond lengths (Å)	0.013	0.002	0.003
RMSD bond angles (°)	1.35	0.43	0.61
MolProbity score	1.17	0.91	2.01
Ramachandran favored/outliers (%)	95.89/0.6	95.90/0.82	95.4/1.6
PDB entry	7Q6K	7Q6J	7YWD

### Modeling, simulation, and bioinformatics

A model for full‐length GDAP1 was obtained from AlphaFold2 [42] and used for further analyses as such. In addition, crystal structure‐based models were prepared and analyzed. Missing loops of the wtGDAP1 crystal structure were built with YASARA [43], and the structure was minimized. The model was further used as a starting point for small‐angle X‐ray scattering (SAXS) data fitting (see below), as well as molecular dynamics (MD) simulations.

MD simulations were run on a GDAP1 monomer, with all loops in place. The simulations were run using GROMACS [44], with input file preparation on CHARMM‐GUI [45]. The force field used was CHARMM36m [46], with a cubic box and a 10‐Å extension around the protein. Solvation was done with the TIP3P water model in 0.15 m NaCl. Temperature (NVT) equilibration to 300 K and pressure (NPT) equilibration, via isotropic pressure coupling, were carried out using the Berendsen thermostat.

Structural properties of GDAP1 were analyzed with bioinformatics tools, including NAPS [47] for centrality analyses and DynaMine [48] for prediction of flexibility. Stability effects of missense mutations were predicted with cupsat [49] and maestro [50, 51]. Hydrophobic clusters were identified with proteintools [52].

### Small‐angle X‐ray scattering

The structure and oligomeric state of the GDAP1 R120W and H123R mutants were analyzed with SEC‐SAXS on the SWING beamline [53] (SOLEIL synchrotron, Saint Aubin, France). Samples were dialyzed against fresh SEC buffer and centrifuged at > 20 000 ***g*** for 10 min at +4 °C to remove aggregates. 50 µL of each protein sample at 8.5–10 mg·mL^−1^ was injected onto a BioSEC3‐300 column (Agilent, Santa Clara, CA, USA) at a 0.2 mL·min^−1^ flow rate. SAXS data were collected at +15 °C, over a *q*‐range of 0.003–0.5 Å^−1^ (*q* = 4π sin(θ)/λ, where 2θ is the scattering angle).

Further processing and modeling were done using atsas 3.0 [54]. Scattering curves were analyzed and particle dimensions determined using primus [55] and gnom [56], respectively. Chain‐like *ab initio* models were generated using gasbor [57]. Different GDAP1 dimer models were fitted in a complementary approach against the experimental SAXS data using CRYSOL [58]. SUPCOMB was used to superimpose SAXS models and crystal structures [59].

### Circular dichroism spectroscopy

Synchrotron radiation circular dichroism (SRCD) spectra were collected from 0.5 mg·mL^−1^ samples on the AU‐CD beamline at the ASTRID2 synchrotron source (ISA, Aarhus, Denmark). The samples were prepared in a buffer containing 10 mm HEPES, pH 7.5, and 100 mm NaF. The samples were equilibrated to room temperature and applied into 0.1‐mm pathlength closed circular quartz cuvettes (Suprasil, Hellma Analytics, Müllheim, Germany). SRCD spectra were recorded from 170 nm to 280 nm at +25 °C. Three repeat scans per measurement were recorded and averaged. The CD spectrum baselines were processed and converted to molar ellipticity using cdtoolx [60].

### Thermal stability

Thermal unfolding of GDAP1 variants in SEC buffer was studied by nanoDSF using a Prometheus NT.48 instrument (NanoTemper, Munich, Germany). Tryptophan fluorescence was excited at 280 nm, and emission was recorded at 330 and 350 nm. The samples were heated from +20 to +90 °C with a heating rate of 1 °C·min^−1^, and changes in the fluorescence ratio (F350/F330) were monitored to determine apparent melting points. The data were analyzed using Origin (OriginLab Corporation, Northampton, MA, USA).

### Cell culture and western blotting

Human skin fibroblast cultures were established from skin biopsies of a healthy donor and a patient with GDAP1 H123R mutation [12, 61]. Written consent for the use of patient material was obtained, and the study was approved by the Coordinating Ethics Committee of the Helsinki and Uusimaa Hospital District.

The purification of rDRG sensory neurons, the generation of lentiviral particles, and their use to overexpress the GDAP1 constructs were done as described [62] for MORC2. All animal work was performed in accordance with the Swedish regulations and approved by the regional ethics review committee Stockholm, Stockholms norra djurförsöksetiska nämnd (N79/15). rDRGs were matured into sensory neurons, and GDAP1 expression was induced by doxycycline‐initiated tight‐TRE promoter expression using the lentivirus system [30].

The protein fractions were isolated from the rDRG and fibroblast cells and membranes using 40 mm HEPES, pH 7.0, 400 mm NaCl, and 1% n‐dodecyl‐β‐d‐maltopyranoside, and the supernatant was clarified by centrifugation at 235 000 *g*, +4 °C. The proteins were separated with 12% SDS‐PAGE under nonreducing conditions.

HEK293T‐D10 cells were used to serve as endogenous control and to test the redox sensitivity of the mammalian‐derived GDAP1 samples. Proteins were isolated from total cell lysate and mitochondrial fraction. The mitochondria were isolated from the cells using linear 15–50% (w/v) sucrose gradient centrifugation. The protein was treated with similar lysis conditions as above, and SDS‐PAGE was performed with and without 192 mm β‐mercaptoethanol.

Proteins were transferred onto 0.22‐µm nitrocellulose membranes with the semi‐dry or wet transfer protocol in TurboBlot^®^‐buffer (Bio‐Rad Laboratories, Inc., Hercules, CA, USA). The membrane was blocked with Tris‐buffered saline, 20 mm Tris/HCl (pH 7.4), 100 mm NaCl, 0.1% v/v Tween‐20 (TBST), and 5% w/v casein (milk powder) and incubated for 2 h at +4 °C. The primary antibody, rabbit anti‐GDAP1 anti‐serum [16], was added at a 1 : 5000 dilution and incubated for 1 h at +4 °C, followed by the secondary antibody for 1 h at +4 °C (anti‐rabbit IgG‐HRP, Promega (Fitchburg, WI, USA) 65‐6120). The Pierce^®^ enhanced chemiluminescence substrate (Thermo Fisher Scientific) was added, and the blot was illuminated using ChemiDoc XRS+ (Bio‐Rad). Tubulin was used as a loading control in all experiments.

### Immunofluorescence microscopy

rDRG cells were fixed with 4% paraformaldehyde in phosphate‐buffered saline, 8 mm Na_2_HPO_4_, 2 mm KH_2_PO_4_, 137 mm NaCl, and 2.7 mm KCl (pH 7.4) (PBS) at +22 °C for 10 min, and washed in PBS. They were then incubated for 1 h at +22 °C in a blocking solution (5% bovine serum albumin, 1% goat serum, and 0.3% Triton X‐100 in PBS) and with primary antibodies overnight at +4 °C (primary Abs: mouse anti‐flag—Sigma F1804; rabbit anti‐NF‐145—Millipore (Darmstadt, Germany) AB1987), followed by washing in PBS. The secondary antibodies were incubated for 45 min at +22 °C (secondary Abs: anti‐mouse 594, Invitrogen A11005; anti‐rabbit 488, Invitrogen A11034). The cells were counterstained with 1 : 10 000 4′,6‐diamidino‐2‐phenylindole (DAPI) in PBS for 5 min at +22 °C. The fixed samples were mounted on coverslips with Vectashield, and images were acquired with a Zeiss LSM700 confocal microscope (Carl Zeiss AG, Jena, Germany).

## RESULTS

### Structural effects of CMT mutations H123R and R120W on GDAP1

**Fig. 1:**
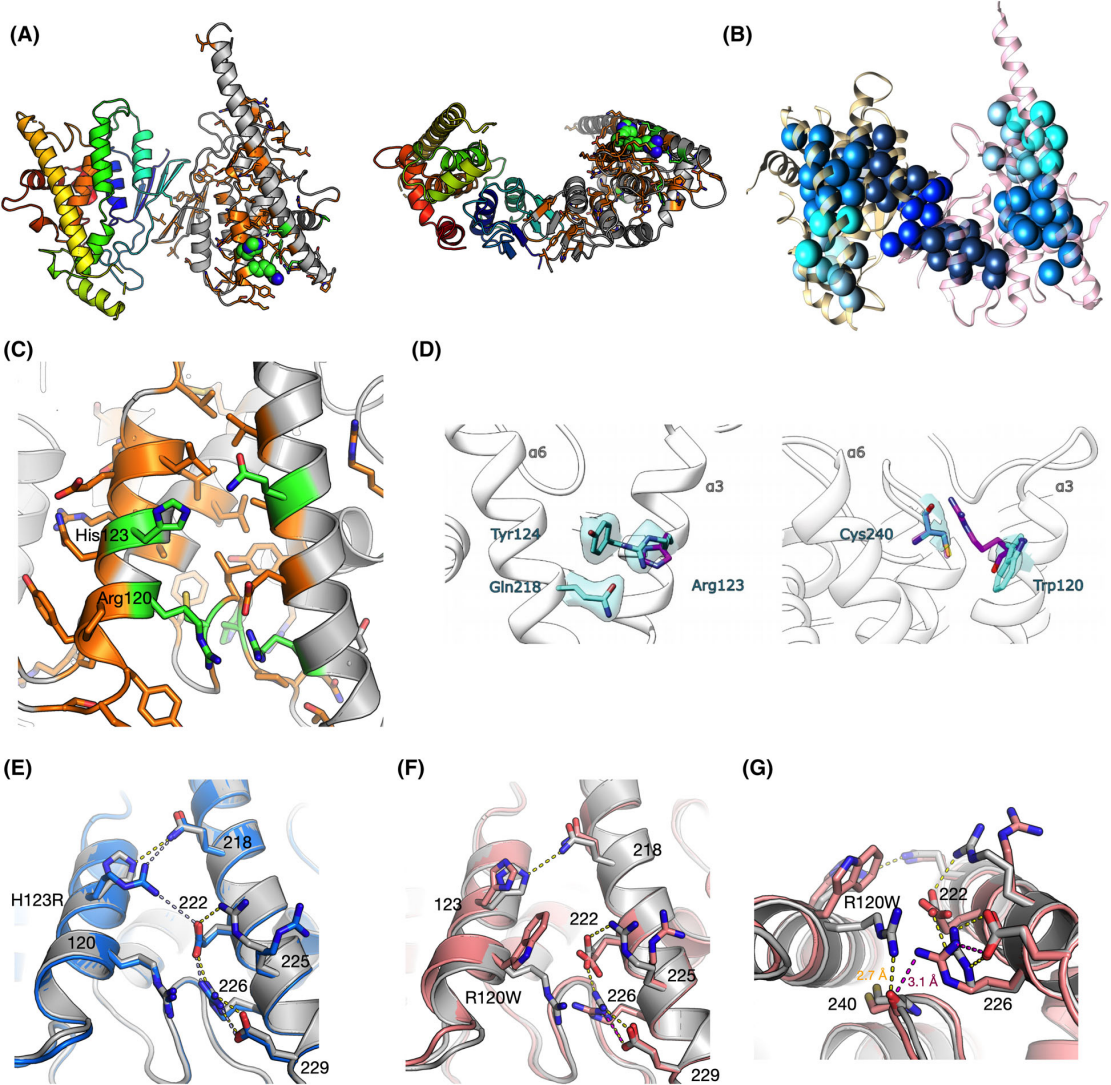
Crystal structure of GDAP1 and effects of CMT mutations. (A) The overall structure of the wtGDAP1 core domain dimer in two orientations, as published before [18]. The left monomer is colored with rainbow colors, while the one on the right is gray and shows the positions of CMT mutations, with side chains visible. The mutations linked to CMT2K are green, while the rest are orange. Arg120 and His123 are shown as spheres. In the right‐hand orientation, the MOM would be below the dimer. (B) Hydrophobic clusters in wtGDAP1. The orientation is the same as the left panel of A, indicating that many CMT mutations lie in close vicinity to the hydrophobic cores. (C) Zoom in on helices ⍺3, ⍺6, and ⍺7. Coloring of CMT mutations is as in A; side chains are shown only for CMT‐linked positions. Note how the CMT‐linked residues participate in a large intramolecular network of interactions. (D) Electron density maps in the mutation sites H123R (left) and R120W (right). 2F_o_‐F_c_ map contours were set to 1.2 rmsd, and the map radius is 1.6 Å from the mutated residue. The corresponding wild‐type residue is shown in magenta in each structure. (E) Comparison between wtGDAP1 (gray) and H123R (blue). Hydrogen bonds are shown as dashed lines. (F) Comparison between wtGDAP1 (gray) and R120W (pink) crystal structures. (G) Interactions around Cys240 in wtGDAP1 (gray) and R120W (pink). Hydrogen bond distances to the backbone carbonyl of Cys240 from Arg120 (wild‐type GDAP1, yellow) and Arg226 (R120W, magenta) are shown.

The CMT‐linked missense mutations in GDAP1 are clustered within the vicinity of the hydrophobic clusters of the N‐terminal GST‐like domain (GSTL‐N), the C‐terminal GST‐like domain (GSTL‐C), and the dimer interface. The affected side chains are often polar or charged and orient toward the solvent (Fig. 1A). They are also close to the hydrophobic clusters of GDAP1 (Fig. 1B). For example, the α6 helix, Lys188‐Glu229, has 20 charged residues along the helix. The clustered mutations could change the side‐chain interaction networks between helices α3, α6, and α7, which further might affect GDAP1 folding and stability. Here, we focused on two CMT‐linked GDAP1 mutations on helix α3 pointing toward α6, R120W, and H123R (Fig. 1A).

We previously determined the crystal structure of wild‐type GDAP1∆303‐358, which corresponds to the GST‐like core domain of GDAP1 in dimeric form, including the GDAP1‐specific insertion [18]. Here, we expressed and purified the variants R120W and H123R, compared them with wild‐type GDAP1 (wtGDAP1) crystal and solution structures, and studied their folding and thermal stability. The crystal of the R120W mutant variant had a new crystal form, while H123R had the same space group as the wtGDAP1 structure, displaying a homodimer in the asymmetric unit. In the H123R structure, the dimer in the crystal is covalently linked via a disulfide bond at Cys88, like the wtGDAP1 protein [18]. The disulfide bridge via Cys88 also exists in the R120W structure, but the dimer is formed via crystallographic symmetry.

Both Arg120 and His123 are on the α3 helix, partially solvent‐accessible (Fig. 1C,D). In both mutant structures, as in wtGDAP1, the most flexible regions are in loops between β3‐β4 at positions Leu71‐Ala77, and α5‐α6 at positions Arg159‐Ile186. The β3‐β4 loop is more structurally ordered in the mutants than in wtGDAP1. The α5‐α6 region corresponds to the GDAP1‐specific insertion in the GST superfamily [63]. In both mutant structures, the flexible loop between helices α6‐α7 is similar to wtGDAP1; the Cα backbone is visible, but side chains have poor density.

In the crystal state, the mutations do not cause major structural changes (Fig. 1D‐F). However, intramolecular interactions are altered. In the H123R structure (Fig. 1E), the His123‐Tyr124 π‐orbital interaction is disturbed in the mutant, while the interaction with the side chain of Gln218 is preserved. The salt bridge network around Glu222 and Arg226 is conserved and now includes Arg123. In chain B, the electron density for Arg123 is weak, indicating the flexibility of the mutant residue.

Arg120 in wtGDAP1 forms a H‐bond with the backbone carbonyl of Cys240, and it is part of a salt bridge network involving Glu222, Arg226, and Glu229 (Fig. 1F). Intriguingly, Arg120 has close contact with Arg226 in wtGDAP1, whereby the two Arg π systems stack, and the surrounding Glu222 and Glu229 neutralize charges via salt bridges. Trp120, as a bulky side chain, causes steric hindrance in the R120W mutant (Fig. 1F), and the α3 helix, carrying Trp120, moves outwards by ~1 Å, and the contact with the neighboring α6 is weakened. The backbone interaction with Cys240 is lost in the mutant, and the salt bridge network centered at Arg226 is disturbed as is the contact between His123 and Gln218, which could be linked to the loss of protein stability. Arg226 turns into the generated pocked, making a weak hydrogen bond to the backbone carbonyl of Cys240 at the expense of its normal interactions (Fig. 1G).

### The mutant proteins show unaltered conformation but lowered stability

**Fig. 2:**
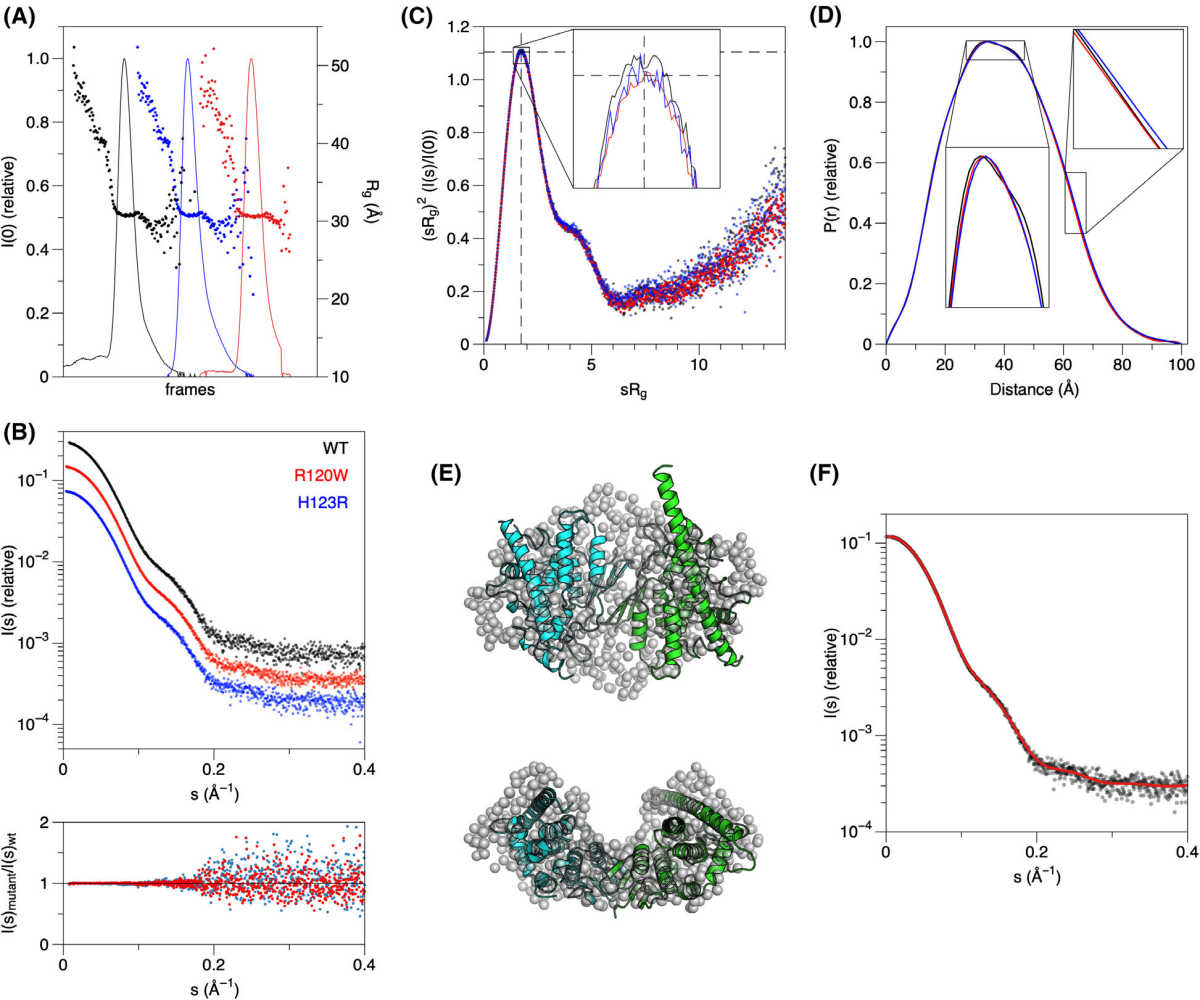
SAXS analysis of the GDAP1 mutations. (A) SEC‐SAXS elution profiles for H123R (blue), R120W (red), and wild‐type GDAP1 (black). The frames from the flat area of R_g_ (dots) in each peak were processed further. The peaks are displaced along the *x*‐axis for clarity. (B) Top: Scattering curves from synchrotron SEC‐SAXS. The curves are displaced in the *y*‐direction for clarity. Bottom: comparison between the mutant data and wtGDAP1 indicates that the SAXS data are essentially identical. (C) Dimensionless Kratky plot shows globular structure and the same level of flexibility. The dashed lines crossing (*x* = √3, *y* = 1.1) reflect the theoretical maximum for a rigid globular particle. (D) Distance distributions indicate similar size and shape, with very minor differences when zoomed in. (E) *Ab initio* chain‐like model (gray spheres) overlaid with the crystal structure of wtGDAP1 [18]. Note how the long helix ⍺6 from the extended conformation does not fit into the envelope. (F) Fit of the *ab initio* model in panel D (red line) to the SAXS data from wtGDAP1 (black dots).

To compare the solution and crystal structures, SAXS analysis was performed on the H123R and R120W mutants (Fig. 2). SEC‐SAXS was employed to achieve better separation between monomer and dimer fractions. Previously, we showed this equilibrium to be concentration‐dependent; high concentration favors the dimeric form [18]. The SEC‐SAXS profiles show that the samples are monodisperse with the same radius of gyration, R_g_, across the peak (Fig. 2A). The molecular weight across the main peak showed that in both mutants, the peak contained a dimeric form similar to wtGDAP1; accordingly, the scattering curves for all variants were essentially identical (Fig. 2B).

Further analysis revealed that the R_g_ values matched the ones for wtGDAP1 (Table 2), indicating similar solution conformation and oligomeric state. Both mutants possess a similar globular fold with essentially the same level of flexibility as wtGDAP1, as demonstrated by the dimensionless Kratky plots (Fig. 2C). Distance distribution functions revealed that the particle dimensions in solution are nearly identical between the mutants and wtGDAP1 (Fig. 2D). Hence, at the resolution of a SAXS experiment, neither mutation caused large‐scale conformational changes.

**Table 2:** SAXS parameters. The values for wtGDAP1 are from our previous study [18].

Construct	GDAP1∆303‐358 H123R	GDAP1∆303‐358 R120W	GDAP1∆303‐358 wt
*R*_g_ (Å) from P(r)	30.74 ± 0.05	30.64 ± 0.05	30.60 ± 0.11
*R*_g_ (Å) from the Guinier plot	30.73	30.64	30.70 ± 0.03
*D*_max_ (Å)	99.9	99.95	99.00
Porod volume estimate, *V*_p_ (Å^3^)	107 157	107 947	105 750
sRg limits	0.24–1.29	0.24–1.30	0.41–1.30
MW from the consensus Bayesian assessment based on SAXS data (kDa)	74.3	74.3	72.4
Calculated monomeric MW from sequence (kDa)	35.1	35.1	35.2
SASBDB entry	SASDND6	SASDNE6	SASDJV8

The dimer–monomer equilibrium in GDAP1 is dynamic, and the dimeric form is favored at high concentrations [18]. At the concentrations and conditions used here, GDAP1 exists as a dimer, as indicated by the SAXS data. Chain‐like *ab initio* models confirmed the observation that both mutants are nearly indistinguishable from dimeric wtGDAP1 (Fig. 2E,F). Thus, there is no indication of effects on the oligomeric status by the two mutations.

The SAXS data indicate a dimeric form for both mutants (Table 2). The ambiguity between the two mutants was estimated with AMBIMETER [64], and H123R and R120W have both similar levels of globularity and stability. These observations are in line with the fairly minor conformational differences in the crystal state, whereby R120W—as a more drastic replacement—led to a small movement of the α3 helix and loss of hydrogen bonding interactions. On the contrary, the only fully monomeric GDAP1 mutant we studied earlier, Y29E/C88A, is more globular than any of the dimeric forms [18].

To further compare the molecules in solution and in the crystal, the crystal structure coordinates were fitted against the SAXS scattering curve (Fig. 2E). Based on the analysis, both mutants adopt the dimer form, having very similar folds in solution as the wtGDAP1. After building in the loops, the dimer structure fits the data better than the crystal structure (see below); hence, the dimer observed in the crystal represents the solution structure, with the addition of the GDAP1‐specific flexible insertion.

**Fig. 3:**
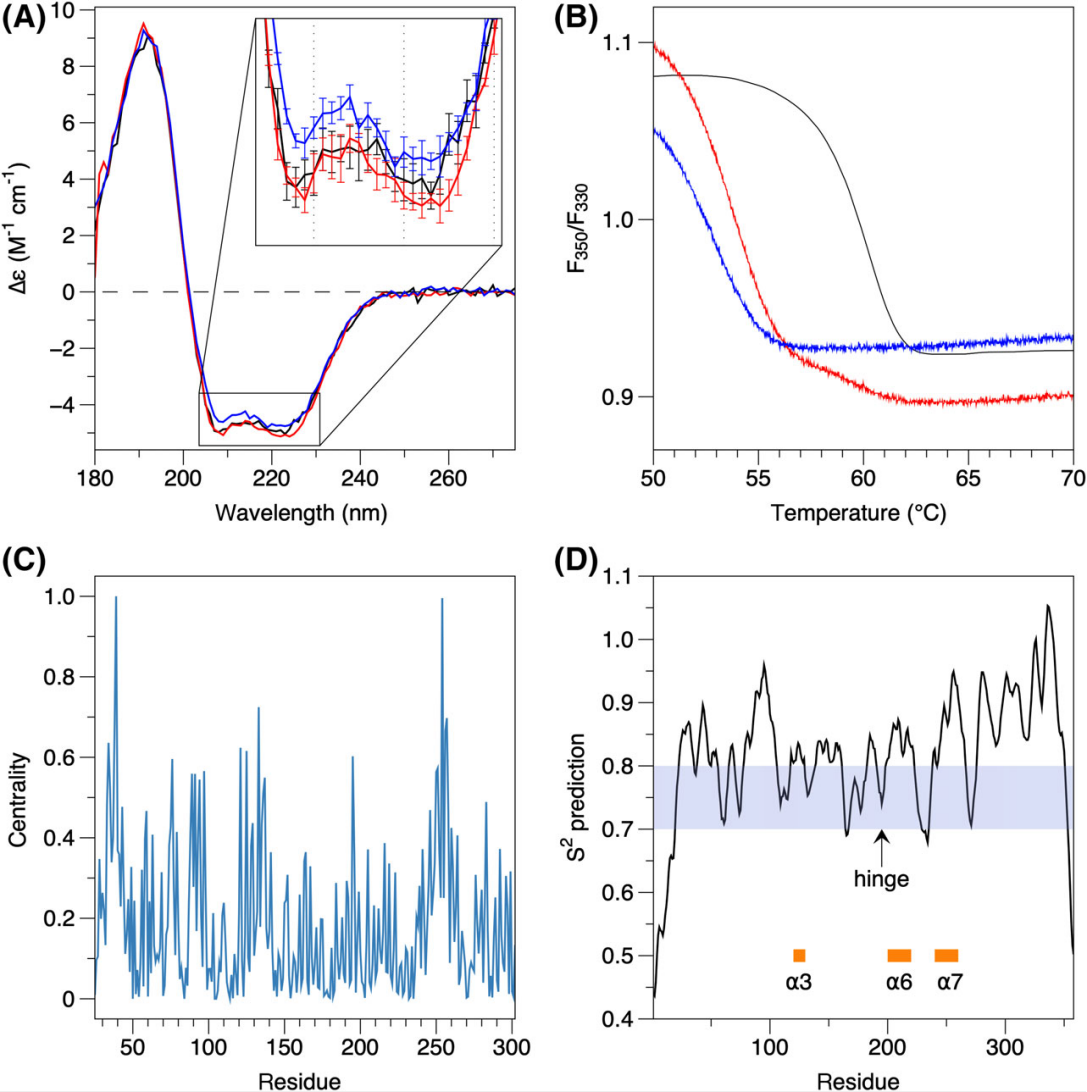
Folding and stability of GDAP1. (A) SRCD spectra for wtGDAP1 (black), R120W (red), and H123R (blue). (B) DSF stability assay. Colors as in A. (C) Residue centrality, as defined by NAPS, identifies helix ⍺7, around residue 250, as the most central part of the structure. (D) DynaMine analysis. The location of the hinge in helix ⍺6 is indicated, as are helices ⍺3, ⍺6, and ⍺7. The shaded region indicates context‐dependent folding, while values above 0.8 predict rigid structure.

Since the mutants presented similar solubility and folding as wtGDAP1, we tested whether the mutations caused changes to GDAP1 stability or secondary structure content. To test for quantitative differences in secondary structures, we measured SRCD spectra (Fig. 3A). The SRCD spectra of the mutants overlay well with the wtGDAP1 CD spectrum, showing that the mutants, on average, have a similar secondary structure composition in solution as wtGDAP1. The CD peak at 208 nm is weaker for H123R, which may indicate minor differences in intramolecular interactions between ⍺‐helices, as seen in the crystal structure. Once the GDAP1 dimer forms via the disulfide bond, the structure becomes very stable, and it is challenging to dissociate the dimer [18]. Due to the high helical content in GDAP1 and the similarity of the CD spectra, we can confirm that the mutations do not affect the overall folding characteristics of GDAP1. Small differences in spectral shape may be caused by both local stacking of amino acid side chains and interactions between secondary structure elements [65, 66].

To determine thermal stability, we studied the R120W and H123R variants using the Trp fluorescence emission peak ratio at wavelengths 350/330 nm in nanoDSF. The wtGDAP1 protein is more stable than the mutants (Fig. 3B). The apparent T_m_ value for wtGDAP1, ~62°C, was >5°C higher than for both mutants, suggesting that the effect of the mutations may be linked to an overall destabilization of the fold. Considering the location of the mutations, a region of GDAP1 is revealed, which is important for protein stability.

To obtain further insight into the effects of the mutations on GDAP1, we used a variety of bioinformatics tools. Analyses of centrality (Fig. 3C) indicated that the core region of GDAP1, close to both Arg120 and His123, with helix ⍺7 the most central element, is likely to be important for folding and stability. Many other CMT mutations cluster into this area [18], affecting a number of residues in an interaction network (Fig. 1C). Effects of point mutations on protein stability against temperature or chemicals were predicted using CUPSAT [49]. Both R120W and H123R are predicted to be destabilizing in both respects, in line with the thermal stability data above. DynaMine analysis (Fig. 3D) of protein flexibility based on sequence data further showed that the part of helix ⍺6 before residue 200 has context‐dependent rigidity, indicating that the unique ⍺5‐⍺6 loop in GDAP1 is structurally dynamic.

To complement the above analysis, we predicted the effects of all possible mutations in human GDAP1 on the protein stability, using the crystal structure of wild‐type GDAP1 [18] as a starting point. Using two alternative prediction algorithms [49, 50, 51], we observed an overall destabilizing effect predicted for the CMT‐linked GDAP1 mutations. Only ~10% of all CMT mutations were predicted to be stabilizing by both programs, and individually, both methods predicted ~70–80% of the known mutations to decrease GDAP1 stability. No clear difference was observed between dominant and recessive mutations in this respect, but the numeric values of ∆∆*G* indicate a slightly stronger overall destabilizing effect for the dominant mutations. The results of the predictions are summarized in Table 3, and the raw data can be found in Data S1.

**Table 3:** Predicted effects of CMT missense mutations on GDAP1 stability, using the CUPSAT and MAESTRO servers. The structure used for the predictions was the A chain of wild‐type GDAP1, PDB entry 7ALM. Only residues present in the crystal structure were considered. Note that for CUPSAT, a negative sign for ∆∆*G* corresponds to destabilization, while a positive ∆∆*G* is destabilizing in MAESTRO.

	All mutations	Autosomal recessive	Autosomal dominant
Fraction of destabilizing mutations (CUPSAT)	69%	68%	60%
Fraction of destabilizing mutations (MAESTRO)	73%	77%	80%
Mutations predicted as destabilizing by both algorithms	53%	58%	50%
Mutations predicted as stabilizing by both algorithms	11%	13%	10%
Average ∆∆*G* (CUPSAT, kcal·mol^−1^)	−0.52	−0.06	−0.60
Average ∆∆*G* (MAESTRO, kcal·mol^−1^)	+0.88	+0.84	+1.13

### Flexibility of the α6 helix

**Fig. 4:**
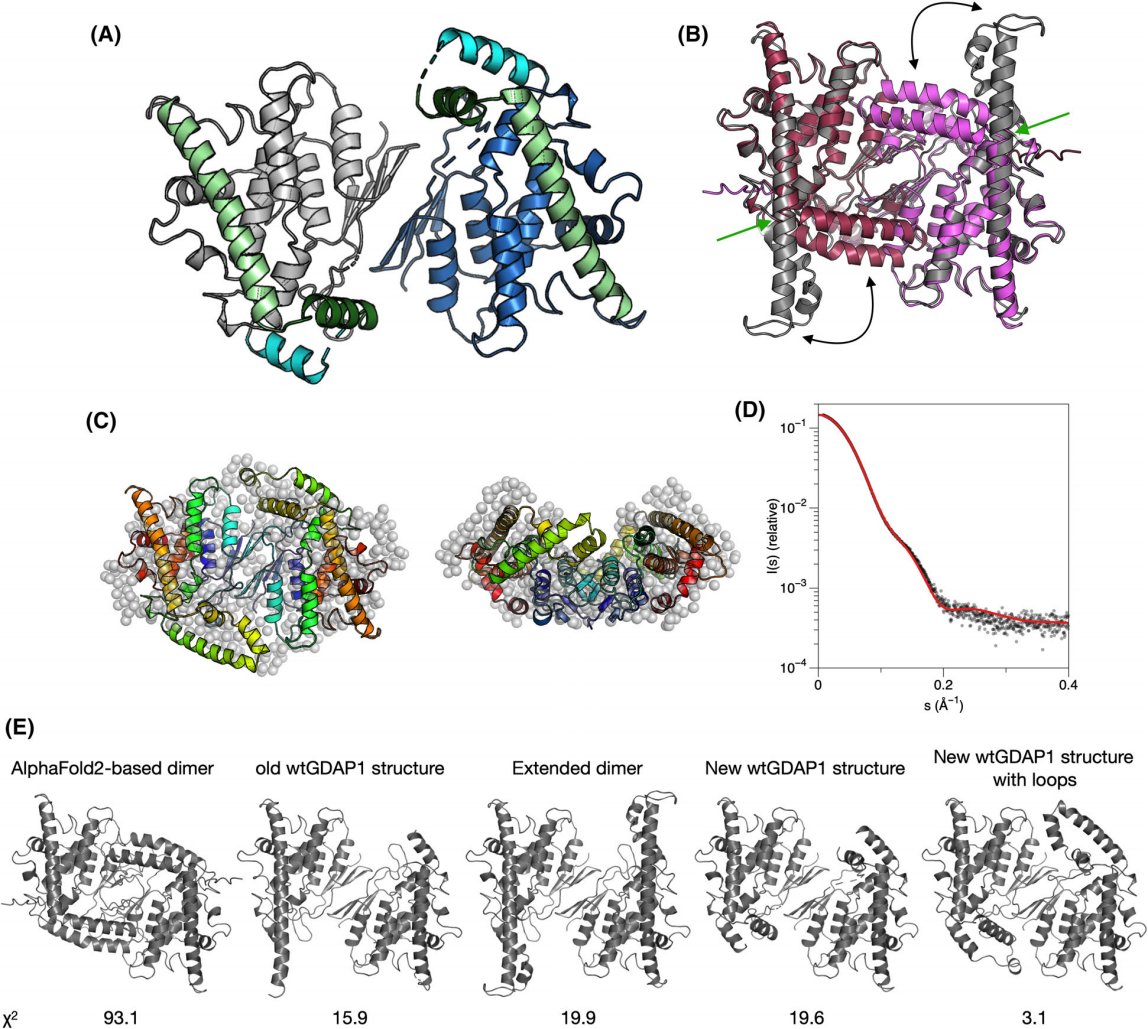
New crystal form of wtGDAP1. (A) The new structure has the long ⍺6 helix divided into two (light and dark green). (B) Comparison of modeled dimers based on the extended crystal form of wtGDAP1 (gray) and AlphaFold2 (red/magenta). The double arrows indicate flexibility of the ⍺5‐⍺6 segment, while the green arrow identifies the hinge in the middle of ⍺6. (C) Superposition of the chain‐like model with the model based on the new crystal structure (cartoons). See further comparisons in panel (E). (D) Fit of the cartoon model from panel C to the wtGDAP1 SAXS data. (E) Fitting of different models to the SAXS data. Shown are models and corresponding chi‐squared values to wtGDAP1 SAXS data. The best fit is obtained with the conformation from the new wtGDAP1 structure, when missing loops are built (see panels C–D).

A crystal structure was solved for wtGDAP1Δ295‐358, and it presented a novel crystal form with four monomers in the asymmetric unit. One complete dimer was present in the asymmetric unit, in addition to two half‐dimers, which both homodimerize through crystallographic symmetry. As the resolution of the structure was rather low, structural details were not analyzed. However, in all four independent protomers, helix α6 breaks in the middle around Asp200, and a helix at residues 189–198 is present in electron density (Fig. 4A). Thus, the long α6 helix can adopt different conformations even in the crystal state. The wtGDAP1 crystal structure published earlier (Fig. 1A) had an asymmetric dimer, with one short and one long α6 helix [18]. These observations suggest that the GDAP1‐specific insertion is also flexible in solution.

The new wtGDAP1 dimer structure was analyzed with respect to the SAXS data, together with a dimer built based on the AlphaFold2 model monomer. The AlphaFold2 model has the helix α6 divided into two and collapsed into a similar, but even more compact, conformation as seen in the new wtGDAP1 crystal (Fig. 4B). However, it is evident from data fitting that the AlphaFold2 model is too compact, while the extended conformation of helix α6 is too elongated. An excellent fit to the SAXS data was obtained using the dimer from the new wtGDAP1 crystal structure with built‐in missing loops (Fig. 4C–E).

**Fig. 5:**
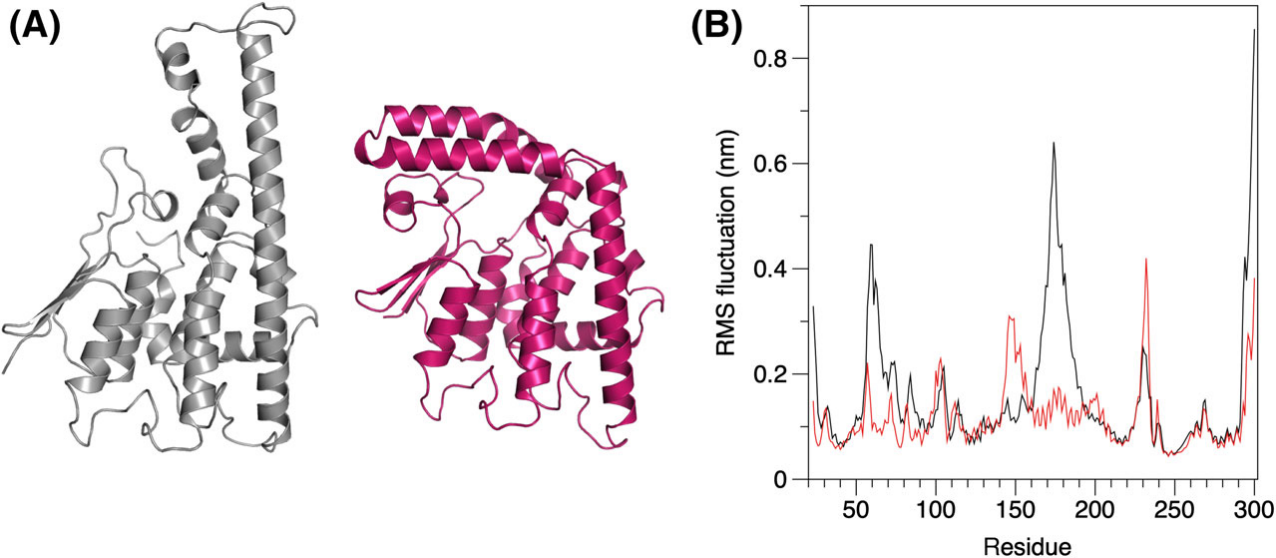
MD simulation of the GDAP1 monomer. (A) The open (gray) and closed (red) conformations of a GDAP1 monomer, obtained from the extended crystal structure and the AlphaFold2 model, respectively. (B) RMS fluctuations of C⍺ atoms indicate relative rigidity of the AlphaFold2 model (red) at the β2‐β3 loop and the ⍺5‐⍺6 loop, compared with wtGDAP1 (black). The simulations were run for 500 ns for the AlphaFold2 model and 350 ns for the open crystal structure model.

To further analyze the dynamics of GDAP1, the model based on the wtGDAP1 crystal structure [18], with all loops added, was subjected to MD simulations (Fig. 5). Throughout the simulation, the GDAP1‐specific insertion is the most dynamic segment of the protein, but the long helix remains extended. On the contrary, simulation of the AlphaFold2 model indicates the stability of the bent conformation. The simulations support the crystal structures of both conformations and give additional proof about a hinge in the middle of helix ⍺6.

### GDAP1 mutant localization and oligomeric state in cells

To explore the two CMT‐linked mutations, R120W and H123R, at the cellular level, we used three different cell culture models, in which either endogenously expressed GDAP1 variants or inducible systems to overexpress GDAP1 variants were utilized.

**Fig. 6:**
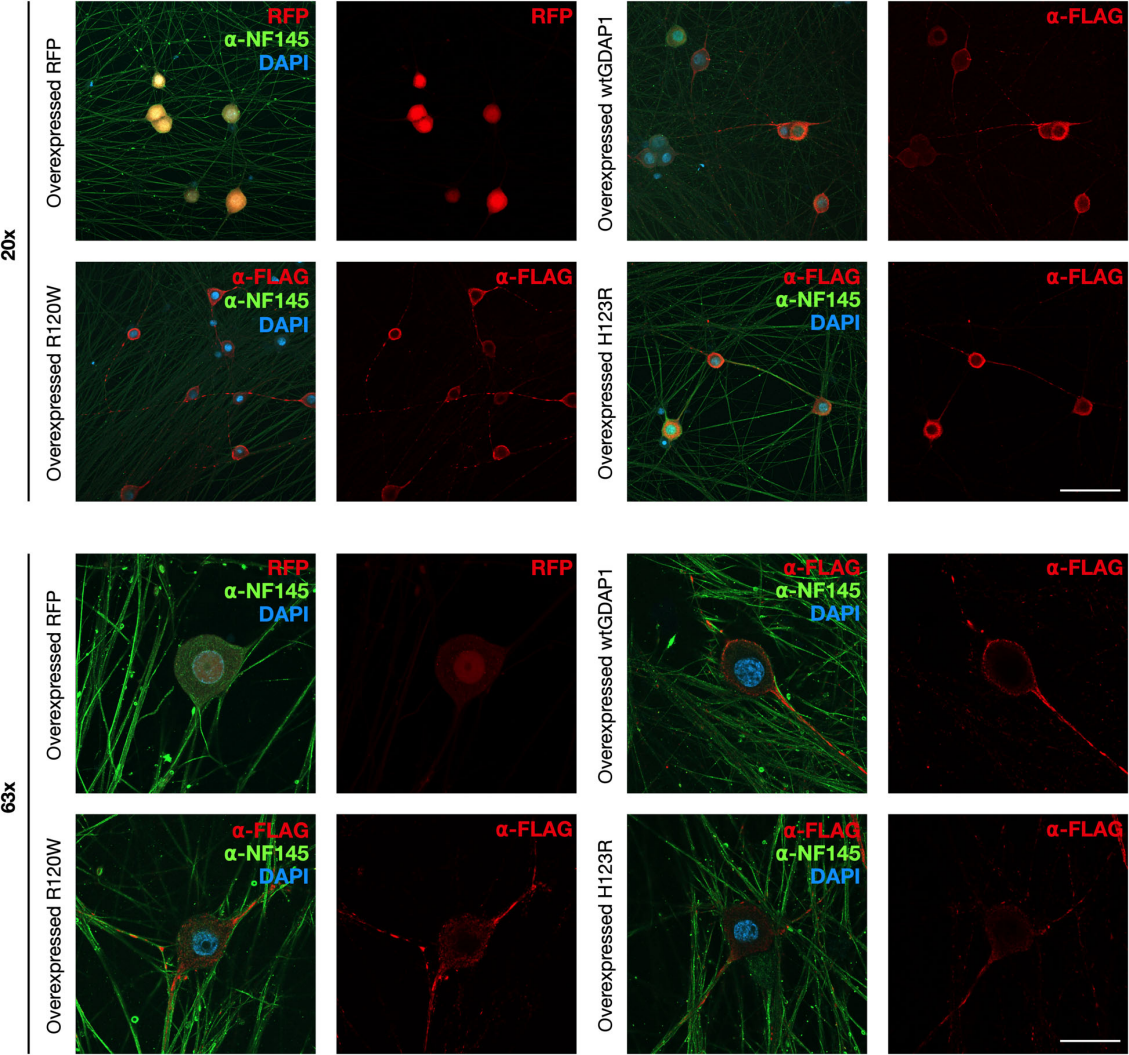
Immunofluorescence analysis of GDAP1‐overexpressing rat DRGs. The images were taken with two magnifications from cells overexpressing wtGDAP1, H123R, R120W, or red fluorescent protein (RFP). GDAP1 staining was done with α‐FLAG, and α‐NF145 was used to visualize neurons. DAPI staining shows the nucleus. Scale bars: 100 µm (top panels, 20×) and 30 µm (bottom panels, 63×).

The localization of R120W and H123R in neurons was compared to that of wtGDAP1 using rDRG primary cell cultures. After protein induction, the neurites were immunostained and imaged using a confocal microscope (Fig. 6). FLAG‐tagged wtGDAP1 locates both in the cell body and in the axons of the sensory neurons. The localization is not cytoplasmic or on the plasma membrane and, in accordance with previous work, most likely mitochondrial. No apparent difference in the immunostaining intensity or cellular localization of R120W or H123R was observed compared with wtGDAP1. None of the mutations induced distinguishable morphological changes in sensory neurons, and no cell toxic effects were observed (Fig. 6).

**Fig. 7:**
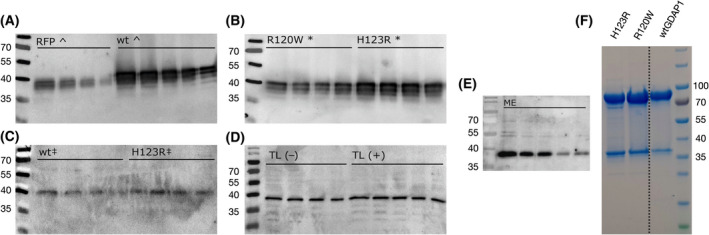
Oligomeric state of GDAP1 in cell lysates. Western blot analysis of rDRG sensory neurons overexpressing RFP and wtGDAP1 (A), as well as R120W and H123R (B). (C) Western blot of human fibroblasts with normal (wt) and disease (H123R) genotype. (D) Total lysate (TL) from HEK293T cells. The blot was run with (+) and without (−) β‐mercaptoethanol in the sample buffer. (E) Mitochondrial extract from HEK293T cells. (F) Purified recombinant GDAP1 soluble GST‐like domain is dimeric under nonreducing SDS/PAGE.

Dimeric GDAP1 has been detectable from mammalian sources [67]. To further test whether full‐length GDAP1 preferably forms disulfide‐linked dimers in cells, we performed western blot analysis for protein extracts under nonreducing conditions (Fig. 7). We analyzed rDRG sensory neurons overexpressing full‐length GDAP1 variants (Fig. 7A,B) and HEK293T‐D10 cells that endogenously express GDAP1 (Fig. 7D,E) to explore the oligomeric state of wtGDAP1, R120W, and H123R. In all cases, nonreducing SDS‐PAGE and western blotting showed only a monomeric form. We then used human fibroblast cultures established from skin biopsies of a CMT2K patient carrying the H123R GDAP1 allele [12, 61]. Normal *GDAP1* genotype fibroblasts were used as control. Western blot analysis revealed that in the fibroblasts, full‐length GDAP1 exists as monomers in both CMT2K patient‐derived cells and healthy control samples (Fig. 7C). For comparison, purified recombinant GDAP1 core domain (lacking the transmembrane domain) and its mutant variants were predominantly dimeric under nonreducing SDS‐PAGE (Fig. 7F).

In all cell lysates studied here, full‐length wtGDAP1, as well as both mutant variants thereof, was detected as a monomer after electrophoresis (Fig. 7). Hence, any dimer present in the cells in these experiments, in the presence of the transmembrane domain, is not disulfide‐linked. Of note, the mutation C88A did not make the recombinant GDAP1 GST‐like domain fully monomeric in our earlier study [18], indicating that the homodimer of the folded core domain can form without the disulfide bridge *in vitro*. The only GDAP1 variant we have observed to be fully monomeric is the double mutant Y29E/C88A [18], disturbing both the hydrophobic interface and the disulfide bridge. To conclude, the main form of full‐length GDAP1 in cells, at least in the absence of inducing factors, is not a disulfide‐linked dimer.

## Discussion

Single‐amino‐acid substitutions can alter protein physicochemical properties, affecting protein stability and function. From a clinical perspective, inherited neuropathies are generally well characterized at the level of the symptomatic spectrum and disease progression. A large variety of CMT mutations are known and characterized clinically [68]. However, in many cases, the molecular basis of these disorders cannot be adequately explained. The difficulty of understanding the mechanism is due to both the vast number of the involved genes and their heterogeneous inheritance patterns and phenotypes, as well as limited knowledge about molecular structure and function.

*GDAP1* is one of the genes associated with peripheral neuropathies caused by missense mutations. We performed structural analyses for two human CMT2K‐linked GDAP1 mutations on helix ⍺3, which revealed that apart from the mutated residue and its immediate surroundings, the overall fold does not change. However, both mutations introduce changes in intramolecular networks and differences in molecular properties, most notably in thermal stability. The structural analysis of pathogenic CMT‐linked GDAP1 variants shows that the mutations are close to the GDAP1 hydrophobic cluster and mediate interactions between key helices of the structure.

### CMT mutations in GDAP1 cluster into hot spots in 3D space

Currently, there are at least 103 *GDAP1* mutations linked to CMT, out of which 68 are reported missense mutations [69]. The functional effects of several GDAP1 mutations have been studied in cells using neurons and Schwann cells or yeast models [7, 10, 70, 71]. As the actual function of GDAP1 at the molecular level, including possible enzymatic activity, remains unclear, additional insights can be obtained using structural biology techniques.

We chose to investigate the Finnish H123R founder mutation, as well as R120W, due to its well‐established clinical and molecular characterization and its location in the vicinity of His123, on helix ⍺3. Both mutations have been linked to the autosomal dominant form CMT2K. Both R120W and H123R are common mutations in European patients [7, 14, 72], and H123R was identified as a major founder mutation in the Finnish population [12, 13].

The GDAP1 crystal structure allows predicting the molecular basis for many of the known mutations in the Human Gene Mutation Database (http://www.hgmd.cf.ac.uk/ac) and the Inherited Neuropathy Variant Browser database (https://neuropathybrowser.zuchnerlab.net). A CMT‐related mutation cluster of GDAP1 (Fig. 1) localizes more on helices α3 and α6 and less on helices α7, α8, and their connecting loops [18]. There are 68 known missense GDAP1 mutations involving 39 residues. The main cluster contains 27 residues that form a network of interactions, including salt bridges, hydrogen bonds, and van der Waals contacts. These interactions provide extensive contacts between helices ⍺3, ⍺6, and ⍺7 (Fig. 1C). Centrality analyses of the GDAP1 structure highlight this, indicating helix ⍺7 as the most central segment of the GDAP1 structure. Notably, many mutations linked to CMT2K map very close to each other in 3D space, suggesting an intramolecular network that may get disturbed upon disease mutations, altering GDAP1 structure or function.

Mutating residues His123 and Arg120 does not break the GDAP1 fold, but rather may affect the intramolecular residue interaction network and protein stability. The thermal stability of the mutant proteins decreased compared with wtGDAP1, suggesting that especially the interaction between helices α3 and α6 may be important. Predictions of ∆∆*G* using computational methods mainly agree with the experiment, suggesting that both mutations are destabilizing; overall, CMT mutations are predicted to destabilize GDAP1 (Table 3). Interestingly, the wedge between helices α5 and α6 contains a pattern of double tyrosines, double glutamates, double leucines, and double lysines, which seem to pull the GSTL‐C core together. The mutations in many cases are introduced into the neighboring positions of these double pairs, like in the case of H123R.

We showed that hexadecanoic acid bound into a groove close to the CMT mutation cluster [18]. The R120W and H123R mutation sites are close to the α6 helix, and the main hydrophobic cluster centered around α7. As an interesting hot spot, Arg120 is the site for four different CMT mutations. Mutations in such clusters and hot spots might affect residue interaction networks and thus decrease protein stability.

### The network of interactions is sensitive toward CMT mutations

Considering the interactions between His123 and Arg120, as well as the networks between helices ⍺3, ⍺6, and ⍺7, it becomes evident that several involved residues are targets of CMT mutations. An example is Glu222, which is sandwiched between three Arg side chains (Arg120, Arg225, and Arg226) and Tyr124, and has van der Waals contacts to Leu239. Another example is Ala247; the conservative mutation to valine is linked to disease [73]. Ala247 on helix ⍺7 is located in a tight space right below His123 and Arg120 and part of the same interaction network. The apparently mild CMT mutation A247V increases the size of the side chain and affects the local interactions. Cys240 from the ⍺6‐⍺7 loop also lies right below His123 and Arg120, and its mutation to Tyr in CMT [70, 74] will interfere with the local interaction network at this residue cluster.

Taken together, although the CMT mutations in GDAP1 initially appear to be scattered throughout the sequence, in the 3D structure, they are often involved in close, complex interaction networks, and these networks are sensitive toward changes in many different participating residues. This observation, together with our bioinformatics analyses, suggests the loss of protein stability upon mutations in such networks and clusters and may hint at an overall mechanism of GDAP1‐linked CMT. Similarly, we earlier observed a decrease in protein stability for all known CMT‐linked mutations in the myelin protein P2 [75, 76]. It should, however, be noted that the exact linkage of the observed decrease in protein stability caused by CMT mutations *in vitro* and *in silico* to actual alterations in GDAP1 protein function *in vivo* remains currently unclear.

**Fig. 8:**
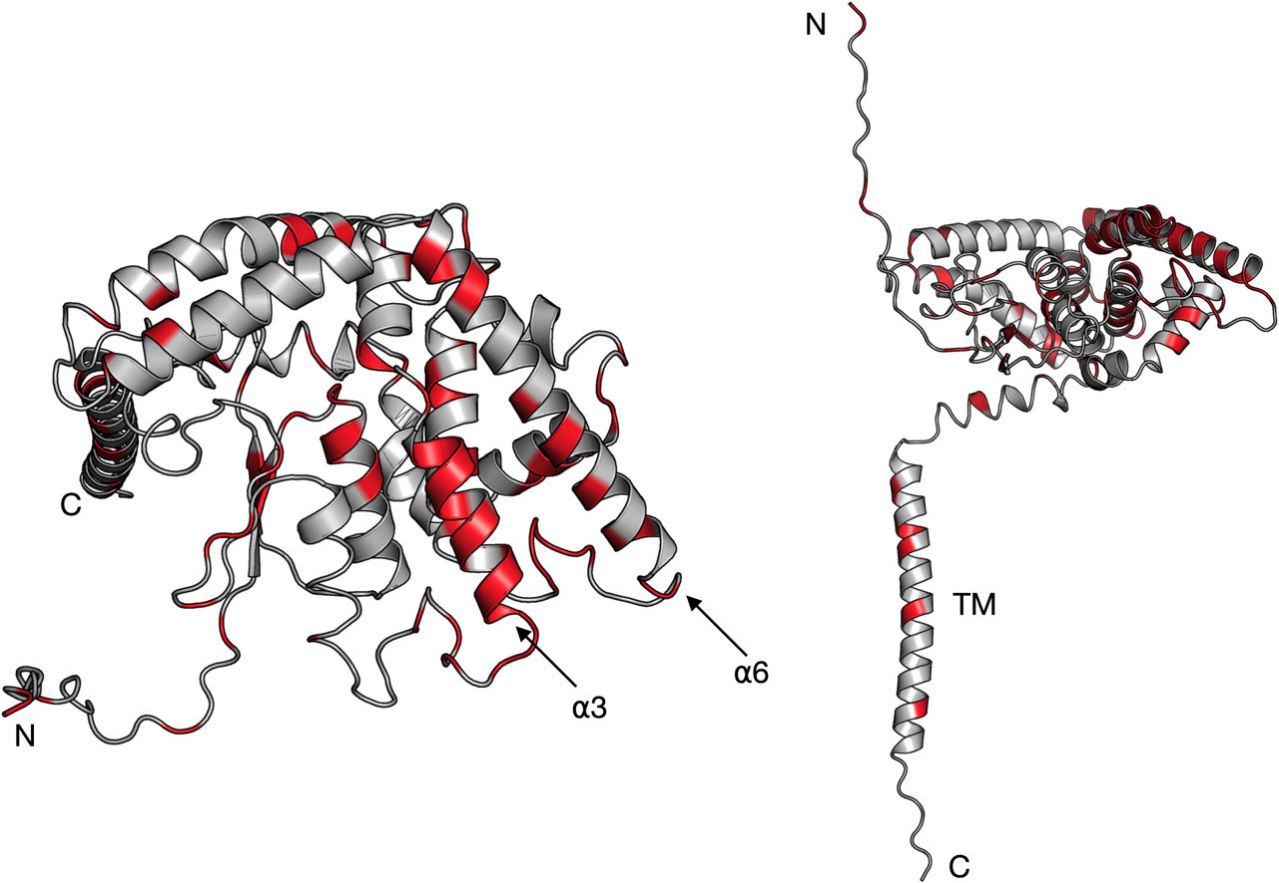
Locations of known CMT mutations in GDAP1. Shown is the AlphaFold2 model of full‐length GDAP1 monomer in two orientations, and the mutation sites are highlighted in red. Note how mutations cluster into the folded GST‐like core domain, especially on the outside of helix ⍺3 and the inside of helix ⍺6. The few mutations in the transmembrane domain (TM) are linked to severe phenotypes.

A key feature of GDAP1—not present in the construct used for structural studies here—is a single transmembrane helix, which anchors into the MOM. The residues linked to the most severe phenotypes of CMT locate in the close vicinity or inside the transmembrane helix [77], hence not participating in the interaction networks within the folded GDAP1 core domain, while the vast majority of the mutation sites cluster into the folded GST‐like domain (Fig. 8). Our data show that the R120W and H123R mutations had only minor effects on the crystal structure, while decreasing the GDAP1 fold stability. Such effects might be altered once the protein is fully constituted into a membrane—a question that could be answered through structural studies on full‐length GDAP1 constructs including the transmembrane domain. The severe CMT mutation G237D results in a mistargeted protein due to the inclusion of a negative charge into the transmembrane helix [77]. Interestingly, Gly‐rich sequences, also called glycine zippers, are often involved in transmembrane helix dimerization [78], and the Gly motif in the transmembrane domain could be involved in GDAP1 dimerization. However, we did not find any disulfide‐linked dimeric GDAP1 in cell lysates, containing the endogenous or overexpressed full‐length GDAP1. Although the functional effects of a missing transmembrane domain or some common missense point mutations have been described [16, 79, 80], more comprehensive modeling [81] coupled with complete structural data from full‐length GDAP1 would better explain the mild phenotype mutations. After optimization of large‐scale production of full‐length recombinant human GDAP1 and its mutant variants, future work on GDAP1 structure will include the above aspects.

### The unique ⍺6 helix of GDAP1

Helix ⍺6 is a dominant and unique feature of the GDAP1 structure, being part of the GDAP1‐specific insertion, together with its preceding loop, which is not visible in electron density. We used a combination of crystal structures and computational models to get further insights into the GDAP1 helix ⍺6 and its dynamics. Our observation of the α6 conformational flexibility may point out mechanistically important functions, which could be linked to the effects of CMT disease mutations on or near the helix.

In the original wtGDAP1 structure [18], we saw a breakdown of noncrystallographic symmetry, as the ⍺6 helix was of different lengths between the two chains in the asymmetric unit. The shorter version of the helix started around residue 200, which is the hinge region in our new wtGDAP1 crystal structure, in which the helix continues in another direction in all four independent protomers in the asymmetric unit. A break of the helix at the same position is also predicted by AlphaFold2. However, the conformation of monomeric GDAP1 in the model is incompatible with the exact model of dimerization we observe in the crystal state, leading to steric clashes. Sequence‐based flexibility analysis points out the region around residues 190‐200 as potentially flexible. The conformational dynamics of the GDAP1‐specific insertion, via a hinge around residue 200, could play a role in its physiological functions and its interactions with other molecules, such as the cytoskeleton, *in vivo*.

### GDAP1 is a dimer in solution but not disulfide‐linked in cells

GDAP1 has a unique dimer interface compared with canonical GSTs [18] and dynamic oligomerization properties [18, 67, 82]. Our results show that GDAP1 dimerization mediated via a disulfide bond can also be observed in the CMT mutant proteins *in vitro*. In the cellular environment, such a disulfide bond could be formed via a folding catalyst or through changes in the redox environment. The latter has been linked to GDAP1 function [83].

An interesting possibility for the dimeric function would be GDAP1 activation by a folding catalyst, affecting interactions with a partner protein, suggesting that the GDAP1 function could be linked to its oligomeric state. Many binding targets have been proposed for GDAP1, such as tubulin and other cytoskeletal components [23, 71]. The GDAP1 disease mutations could, in addition to affecting protein folding and stability, modulate protein–protein interactions. However, the details of molecular interactions formed by GDAP1 remain unknown, and further studies should be focused on studying GDAP1–cytoskeleton interactions and their links to GDAP1 oligomeric state.

## Conclusions

We have presented a structural analysis of two CMT‐linked mutations in GDAP1, R120W, and H123R. The effects of these mutations on protein structure were small, and the mutations may affect dynamic properties, stability, and conformational changes of GDAP1 and/or its interactions with additional binding partners. The cluster of CMT‐related mutations in the 3D structure of GDAP1 highlights a tightly interwound network of amino acid side‐chain interactions that are likely essential for the normal function and structure of GDAP1. Such mutation clustering establishes the need for accurate structural studies of proteins targeted by disease mutations, and it can be expected that most of the mutations in such a cluster similarly affect protein stability or functional interaction networks. A major goal for the future will be the structure solution of full‐length GDAP1 protein, including the transmembrane domain, in addition to deciphering details of its physiological function and molecular interactions.

## Conflict of interest

The authors declare no conflict of interest.

## Author contributions

AS, GN, AR, LB, HT, and PK conceived of the study. AS, GN, GM, AR, and LB carried out experiments. LB, EY, HT, and RL provided materials and resources. AS, GN, AR, LB, and PK analyzed the data. AS and PK wrote the manuscript. SR, PK, and HT supervised the study. PK acquired funding.

## Supporting information


**Data S1**. Raw results from a full screen of all possible mutations using CUPSAT and MAESTRO. Included are all GDAP1 mutations with a literature reference. The first sheet has all analyzed mutations, and dominant and recessive mutations have their own sheets. This file is in Excel format.

## Data Availability

The crystal structures and the structure factors have been deposited at the PDB with entry codes 7Q6K (R120W), 7Q6J (H123R), and 7YWD (new crystal form of wtGDAP1). The SAXS data for the GDAP1 mutants are available at the SASBDB under entries SASDND6 (H123R) and SASDNE6 (R120W). The diffraction datasets for the mutants were uploaded on Zenodo: https://doi.org/10.5281/zenodo.4686880 (R120W) and https://doi.org/10.5281/zenodo.4686876 (H123R).
